# Postoperative Antimicrobial Prophylaxis Use and Outcomes in Pectus Excavatum Repair

**DOI:** 10.1001/jamanetworkopen.2025.30449

**Published:** 2025-09-04

**Authors:** Kerri A. McKie, Anoosha Moturu, Dionne A. Graham, Melvin Coleman, Reiping Huang, Catherine Grant, Jacqueline M. Saito, Bruce L. Hall, Robert A. Cina, Jason G. Newland, Michael J. Goretsky, Clifford Y. Ko, Shawn J. Rangel

**Affiliations:** 1 Department of Surgery, Boston Children’s Hospital, Harvard Medical School, Boston, Massachusetts; 2 Division of Research and Optimal Patient Care, American College of Surgeons, Chicago, Illinois; 3 Program for Patient Safety and Quality, Boston Children’s Hospital, Boston, Massachusetts; 4 Children’s Surgery Program, Continuous Quality Improvement, American College of Surgeons, Chicago, Illinois; 5 Division of Pediatric Surgery, Children’s National Hospital, Washington, DC; 6 University of California, Davis Health System, Sacramento; 7 Department of Surgery, The Medical University of South Carolina, Charleston; 8 Division of Infectious Disease, Nationwide Children’s Hospital, Columbus, Ohio; 9 Department of Surgery, Children’s Hospital of the King’s Daughters, Norfolk, Virginia

## Abstract

**Importance:**

Postoperative antimicrobial prophylaxis (PAP) is frequently used following pectus excavatum repair and accounts for the highest relative burden of potentially avoidable postoperative antibiotic days among pediatric general surgical procedures.

**Objective:**

To evaluate the association of postoperative antibiotic prophylaxis with postoperative rates of surgical site infections, reoperation, and readmission in children undergoing pectus excavatum repair who did and did not receive postoperative antibiotic prophylaxis.

**Design, Setting, and Participants:**

This cohort study included children aged younger than 18 years undergoing pectus excavatum repair from January 2021 to December 2023 at 141 hospitals participating in the National Surgical Quality Improvement Program–Pediatric.

**Exposures:**

Continuation of prophylactic antibiotics after incision closure.

**Main Outcomes and Measures:**

Primary outcomes included 30-day postoperative rates of surgical site infections (SSIs), reoperation, and readmission. Propensity score matching was used to balance groups (without PAP or with PAP) on patient and operative characteristics plausibly associated with PAP use and outcomes. Mixed-effects models were used to compare outcomes between matched groups, with a random effect used to account for hospital clustering. A complementary hospital-level analysis was used to explore the correlation between rates of PAP use and observed to expected (O/E) rate ratios for each outcome after adjusting for differences in patient and procedural characteristics among hospitals.

**Results:**

A total of 3552 patients were included (median [IQR] age, 15.4 [14.5-16.4] years; 3099 males [87.3%]). Postoperative prophylaxis was used in 1949 patients (54.9%) for a median (IQR) duration of 21.6 (14.6-24.0) hours. In the matched cohort of 3168 patients, outcomes were similar in children receiving PAP compared with those who did not, with 26 of 1584 (1.6%) of children who received PAP developing an SSI compared with 29 of 1584 (1.8%) of children who did not receive PAP (odds ratio [OR], 0.90; 95% CI, 0.50-1.61). For children requiring reoperation, 25 of 1584 (1.6%) received PAP compared with 27 of 1584 (1.7%) who did not (OR, 0.98; 95% CI, 0.52-1.83). For readmission, 41 of 1584 (2.6%) children received PAP compared with 57 of 1584 (3.6%) who did not (OR, 0.81; 95% CI, 0.48-1.34). In the hospital-level analysis, no correlation was found between rates of postoperative prophylaxis use and O/E rate ratios for SSIs (Spearman ρ, −0.07; *P* = .43), reoperation (Spearman ρ, −0.01; *P* = .93), or readmission (Spearman ρ, −0.03; *P* = .65).

**Conclusions and Relevance:**

In this cohort study of 3552 pediatric patients undergoing pectus excavatum repair, postoperative antibiotic use was not associated with improved outcomes, challenging the routine use of postoperative prophylaxis in the operative management of this condition.

## Key Points

**Question:**

In children undergoing minimally invasive pectus excavatum repair, is the use of postoperative antimicrobial prophylaxis associated with improved outcomes?

**Findings:**

In this propensity score–matched multicenter cohort study of 3552 children from 141 hospitals, use of postoperative prophylaxis was not associated with a reduction in rates of surgical site infection, reoperation, or hospital readmission.

**Meaning:**

In this study, postoperative continuation of antimicrobial prophylaxis was not associated with improved outcomes and should be reconsidered in children undergoing minimally invasive pectus excavatum repair.

## Teaser

This cohort study assesses the association of postoperative antibiotic prophylaxis with surgical site infections and other postoperative outcomes in children undergoing minimally invasive pectus excavatum repair.

## Introduction

Pectus excavatum is the most common congenital chest wall deformity, affecting approximately 1 in 400 births.^1,2,3^ In children undergoing operative correction, preoperative antimicrobial prophylaxis is generally recommended due to the implantation of prosthetic material. However, current multidisciplinary consensus guidelines advise against the routine use of postoperative prophylaxis for clean wound class procedures.^4^ Despite these recommendations, postoperative prophylaxis remains widely used among pediatric surgeons, reflecting substantial practice variation.^5,6,7^ This variation is likely driven by the absence of high-quality comparative effectiveness data evaluating the implications of postoperative antibiotic use for surgical site infection (SSI) outcomes in this population.

Given the clinical significance of SSIs and the broader public health concerns related to antimicrobial overuse and resistance, defining the appropriate duration of antibiotic use following surgery is critical. This question is particularly relevant for pectus excavatum repair, which has been shown to contribute disproportionately to potentially avoidable postoperative antibiotic treatment days within pediatric general surgery.^5,8,9^ To address this evidence gap, we conducted a multicenter analysis to evaluate the association between postoperative antimicrobial prophylaxis and the incidence of SSI and other resource-intensive outcomes in children undergoing minimally invasive pectus excavatum repair with or without postoperative antibiotic prophylaxis.

## Methods

### Study Design and Data Source

This retrospective multicenter cohort study of patients who underwent minimally invasive pectus excavatum repair analyzed outcome and prophylaxis use data from the American College of Surgeons’ National Surgical Quality Improvement Program-Pediatric (ACS NSQIP-Pediatric). Antimicrobial prophylaxis data collected by NSQIP–Pediatric includes antibiotics used, timing of administration, and duration of use.^10,11^ All NSQIP-Pediatric data are collected by dedicated surgical clinical reviewers using standardized definitions and data abstraction methods. Accuracy and integrity of the data are ensured through periodic auditing, mandatory recertification of data abstractors, and availability of ACS clinical support personnel to address questions regarding data definitions and the medical record abstraction protocol.^10^ This study was considered minimal risk and, therefore, deemed exempt from review and the informed consent requirement by the institutional review board of Boston Children’s Hospital because it was not human participant research. The Strengthening the Reporting of Observational Studies in Epidemiology (STROBE) guideline for reporting was followed.

To provide complementary insight, we performed analyses at both the patient and hospital levels to examine the association between prophylaxis use and postoperative outcomes. The patient-level analysis assessed whether children who received postoperative antibiotics experienced improved outcomes compared with those who did not, accounting for differences in patient and operative characteristics between groups. The hospital-level analysis evaluated whether children treated at hospitals with higher rates of postoperative antibiotic use had improved outcomes compared with those treated at hospitals with lower rates of use.

### Study Cohort

Children younger than 18 years of age who underwent minimally invasive pectus excavatum repair (*Current Procedural Technology* codes 21742 and 27143) from January 1, 2021, to December 31, 2023, at the 141 participating NSQIP-Pediatric hospitals were identified. Patients were excluded for age older than 18 years, missing prophylaxis or outcomes data, and if concurrent procedures were performed during the operative encounter other than those associated with pain management (eg, cryoablation, injection of neurolytic agents, intercostal nerve blocks) or tube thoracostomy.

### Classifications of Exposures and Outcomes

The primary exposure was the continuation of prophylactic antibiotics after incision closure. The primary outcomes included 30-day rates of postoperative SSIs (incisional or organ space SSIs, as defined by NSQIP-Pediatric), reoperation, and hospital readmission. All outcomes included in NSQIP-Pediatric (including SSI) are identified by dedicated, ACS-trained surgical clinical reviewers (SCRs) using standardized clinical and laboratory-based criteria. This process includes manual medical record review of the index admission and subsequent hospital and ambulatory visits, and outreach to caregivers to assess events that may have been identified at hospitals outside the NSQIP-Pediatric hospital network. Additionally, all readmission and reoperation events occurring within 30 days postoperatively are manually reviewed by SCRs to determine the primary indication for the event, including whether the encounter was related to an SSI.^10^

### Statistical Analysis

In the primary patient-level analysis, propensity score matching was used to balance the comparison groups. A logistic regression model was used to calculate propensity scores for each patient using patient demographics (age, race or ethnicity, American Society of Anesthesiologists physical classification grade, body mass index, asthma, chronic lung disease, and cardiac risk factors), and procedural characteristics (operative time). Race and ethnicity categories included Black, Hispanic, White, unknown or other and were identified by the NSQIP-Pediatric SCRs at each hospital through review of the patient medical record. Other race is defined by NSQIP-Pediatric as American Indian or Alaska Native, Native Hawaiian or Other Pacific Islander, or race combinations with low frequency (this is defined by NSQIP-Pediatric as being assigned to patients in which multiple race options were selected and the combinations had fewer than 50 cases). All covariates, including race and ethnicity data, were analyzed to balance groups on the available covariates plausibly related to either a higher likelihood of receiving postoperative prophylaxis or developing an SSI. Missing body mass index data were handled using multiple imputation (5 imputations) prior to generating the propensity score model. After propensity scores were calculated, children were matched 1:1.

For each imputation, the association between outcomes and the use of prophylaxis was estimated using logistic regression models, with a random effect by hospital used to control for hospital-level clustering. Measures of association were reported as adjusted odds ratios (AORs), and estimates across imputations were combined using multiple imputation techniques.

The balance of the covariates between treatment groups in both the unmatched and matched cohorts was assessed using standardized differences, with an absolute value less than 0.1 indicating adequate balance. The balance of the unmatched and matched distributions of propensity scores and continuous covariates was assessed via graphical methods. Standard summaries to verify the validity of the propensity score model, including the distribution of the propensity scores and summaries of the propensity scores by group, were calculated (eFigure in Supplement 1).

The hospital-level analysis explored the correlation between hospitals’ postoperative antibiotic use and outcomes after adjusting for differences among hospitals in the same patient and procedural characteristics described in the patient-level analysis. Separate Spearman ρ correlations were calculated for 2 measures of hospitals’ use of postoperative antibiotics: rate of use and median duration of use. Adjusted, hospital-level O/E ratios were estimated for each outcome by exponentiating the shrinkage estimate of each hospital’s random effect from a mixed-effects regression model.

Analyses were performed with SAS statistical software, version 9.4 (SAS Institute). The threshold for statistical significance was defined as a 2-sided *P* < .05.

## Results

**Figure 1.**
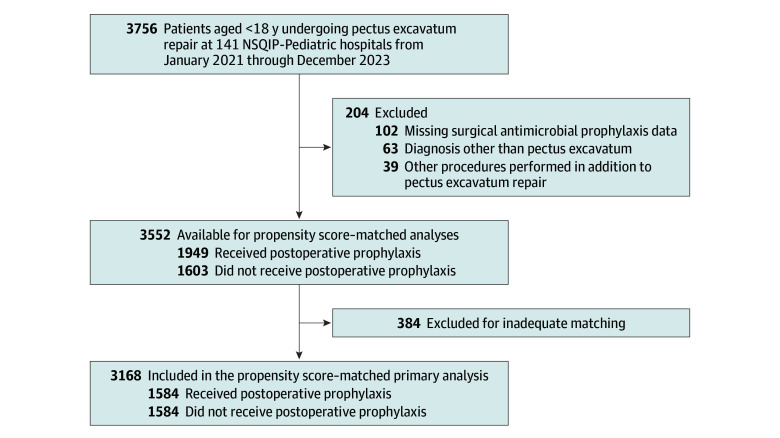
Participant Flow Diagram NSQIP indicates the National Surgical Quality Improvement Program.

In total, 3756 patients undergoing minimally invasive pectus excavatum repair from 141 hospitals were identified. Overall, 204 (5.4%) of the original cohort were excluded from the analysis, leaving 3552 patients in the final cohort (Figure 1). The median (IQR) age at time of operation was 15.4 (14.5-16.4) years, 453 (12.7%) were female, 3099 (87.3%) were male, 51 (1.4%) were Black individuals, 591 (16.6%) were Hispanic individuals, 2508 (70.6%) were White individuals 145 (4.1%) were individuals of other race, and 257 (7.2%) individuals were of unknown race and ethnicity. Of these patients, 241 (6.4%) had a pulmonary comorbidity, and 361 (9.6%) had NSQIP-defined cardiac risk factors (Table).

**Table.** Patient and Operative Characteristics in Children Undergoing Minimally Invasive Pectus Excavatum Repair Who Did and Did Not Receive Postoperative Antibiotic Prophylaxis Before and After Propensity Score Matching

Characteristic	Unmatched cohort			Matched cohort^a^		
	Without antibiotics (n = 1603)	With antibiotics (n = 1949)	Absolute standardized mean difference^b^	Without antibiotics (n = 1584)	With antibiotics (n = 1584)	Absolute standardized mean difference^b^
Age at operation, median (IQR), y	15.5 (14.5-16.5)	15.3 (14.4-16.4)	0.10	15.5 (14.5-16.5)	15.4 (14.5-16.5)	0.00
Sex, No. (%)						
Female	187 (11.7)	266 (13.6)	0.06	186 (11.8)	198 (12.5)	0.02
Male	1416 (88.3)	1683 (86.4)		1398 (88.2)	1386 (87.5)	
Race and ethnicity, No. (%)						
Black	20 (1.3)	31 (51)	0.03	20 (1.3)	22 (8.6)	0.01
Hispanic	277 (17.3)	314 (16.1)	0.03	273 (17.2)	273 (17.2)	0.00
White	1143 (71.3)	1365 (70.0)	0.03	1131 (71.4)	1124 (71.0)	0.01
Other^c^	49 (3.1)	96 (4.9)	0.10	48 (3.0)	53 (3.3)	0.02
Unknown	114 (7.1)	143 (7.3)	0.01	112 (7.1)	112 (7.1)	0.00
ASA grade, No. (%)^d^						
1	357 (22.3)	406 (20.8)	0.04	351 (22.2)	354 (22.4)	0.00
2	1131 (70.6)	1422 (73.0)	0.05	1126 (71.1)	1129 (71.3)	0.00
3	114 (7.1)	120 (6.2)	0.04	106 (6.7)	3.2 (6.3)	0.02
4	799 (0.9)	21 (0.7)	NA	0	0	NA
Unknown	1 (0.1)	1 (0.1)	0.00	1 (<0.1)	1 (<0.1)	NA
Comorbidity, No. (%)						
Asthma	85 (5.3)	91 (4.7)	0.03	80 (5.1)	82 (5.2)	0.01
History of chronic lung disease	10 (0.6)	10 (0.5)	0.01	9 (0.6)	8 (0.5)	0.01
Structural pulmonary abnormalities	19 (1.2)	26 (1.3)	0.01	19 (1.2)	14 (0.9)	0.03
Cardiac risk factors						
Minor risk factors	72 (4.5)	92 (4.7)	0.02	72 (4.6)	71 (4.5)	0.00
Major risk factors	93 (5.8)	104 (5.3)	0.02	91 (5.7)	93 (5.9)	0.01
Operative time, median (IQR), min	111 (86-142)	115 (89-141)	0.01	111 (86-143)	114 (88-140)	0.01

Abbreviations: ASA, American Society of Anesthesiologists; NA, not applicable.

^a^ A total of 141 hospitals were included in the matching.

^b^ Covariates were considered adequately balanced between groups if the absolute standardized mean difference was less than 0.1.

^c^ Other race is defined as American Indian or Alaska Native, Native Hawaiian or Other Pacific Islander, or race combinations with low frequency (defined as multiple race options were selected and the combinations had fewer than 50 patients).

^d^ The ASA physical classification system is a grading system that determines the health of a person before a surgical procedure. Grades range from 1 to 4, with higher grades corresponding to patients with severe systemic disease, functional limitations, and indicating a greater risk of complications or mortality.

Prior to matching, children who received postoperative antibiotics were more likely to be younger than those who did not receive postoperative antibiotics (Table). After matching, children who did not receive postoperative antibiotics were similar to those who did in age as well as all other patient and operative characteristics.

### Surgical Antimicrobial Prophylaxis Use

**Figure 2.**
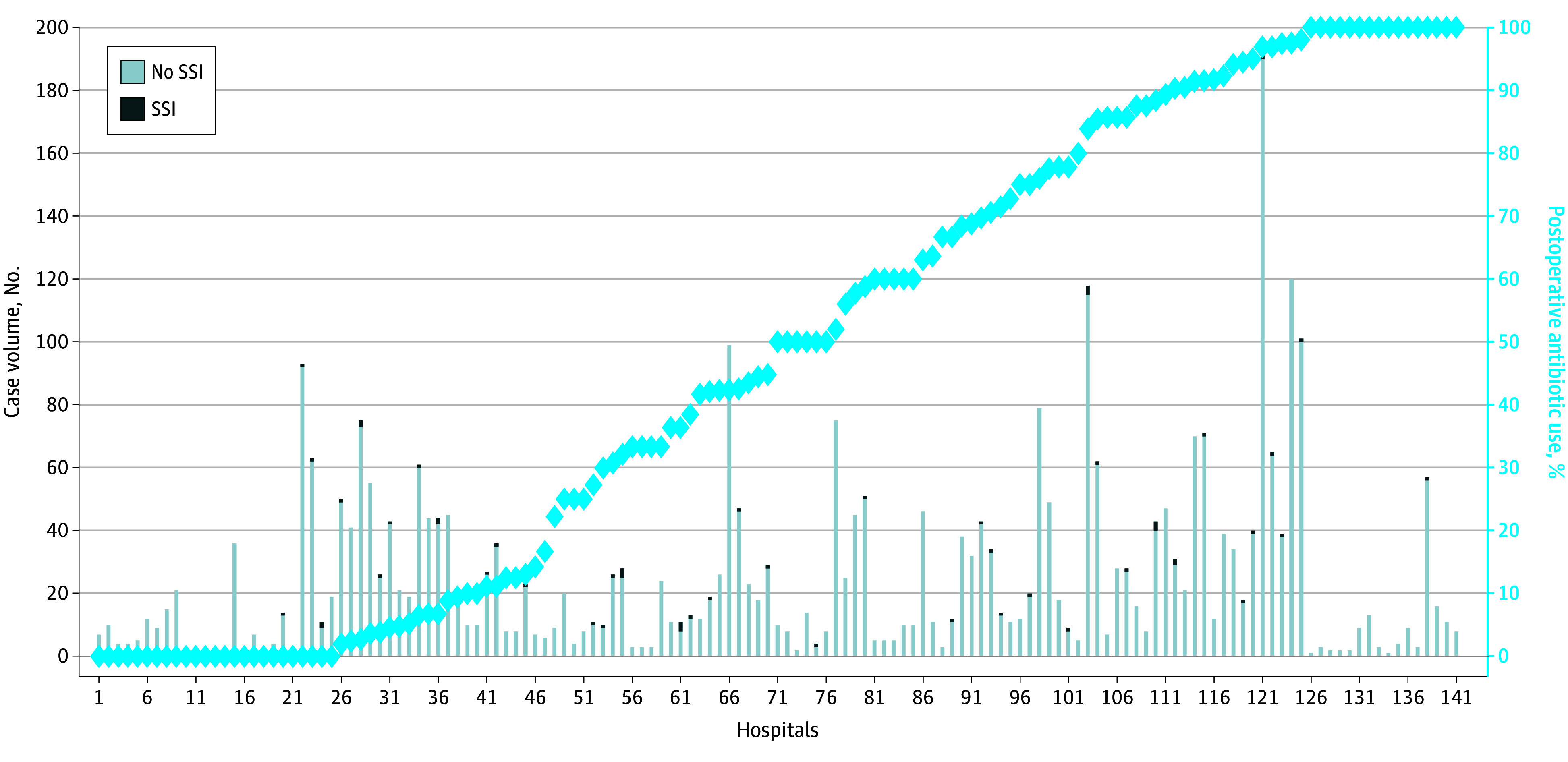
Variation in Postoperative Antibiotic Use and Distribution of Surgical Site Infections (SSIs) in 3552 Children Undergoing Minimally Invasive Pectus Excavatum Repair at 141 Hospitals Participating in the National Surgical Quality Improvement Program–Pediatric Bars represent each hospital included in the study.

All 3552 patients received prophylaxis prior to incision. The most common antibiotics used for prophylaxis included cefazolin (3407 [95.9%]), clindamycin (110 [3.1%]), and vancomycin (20 [0.6%]). After incision closure, 1949 patients (54.9%) received postoperative prophylaxis with a median (IQR) duration of 21.6 (14.6-24.0) hours. Postoperative antibiotic use ranged from 0% to 100% among hospitals (Figure 2). Postoperative antibiotics were used in all patients in 16 of 141 hospitals (11.1%), and in no patients in 25 hospitals (17.7%).

### Infectious Complications

**Figure 3.**
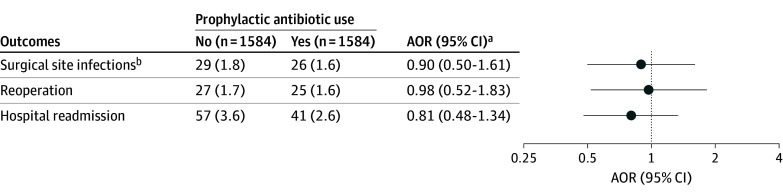
Association of Postoperative Antibiotic Use With Outcomes in Children Undergoing Minimally Invasive Pectus Excavatum at 141 Hospitals Participating in the National Surgical Quality Improvement Program–Pediatric AOR indicates adjusted odds ratio. ^a^Logistic regression was used to explore the association between use of prophylaxis and outcomes in the propensity-matched cohort. ^b^All surgical site infections including superficial, deep, and organ space infections.

The overall postoperative SSI rate was 1.7% (59 of 3552 patients) (Figure 2). Before matching, SSI rates were similar in children who received postoperative antibiotics (30 of 1949 patients, [1.5%]) compared with those who did not (29 of 1603 patients, [1.8%]) (OR, 0.85; 95% CI, 0.49-1.45). In the propensity-matched cohort that included 1584 patients in each group, the overall SSI rate was 1.7%, and SSI rates were similar in the 26 children who received postoperative antibiotics (1.6%), compared with the 29 children who did not (1.8%) (AOR, 0.90; 95% CI, 0.50-1.61) (Figure 3). Postoperatively, 1 patient (0.03%) in the matched cohort developed *Clostridium difficile* colitis.

**Figure 4.**
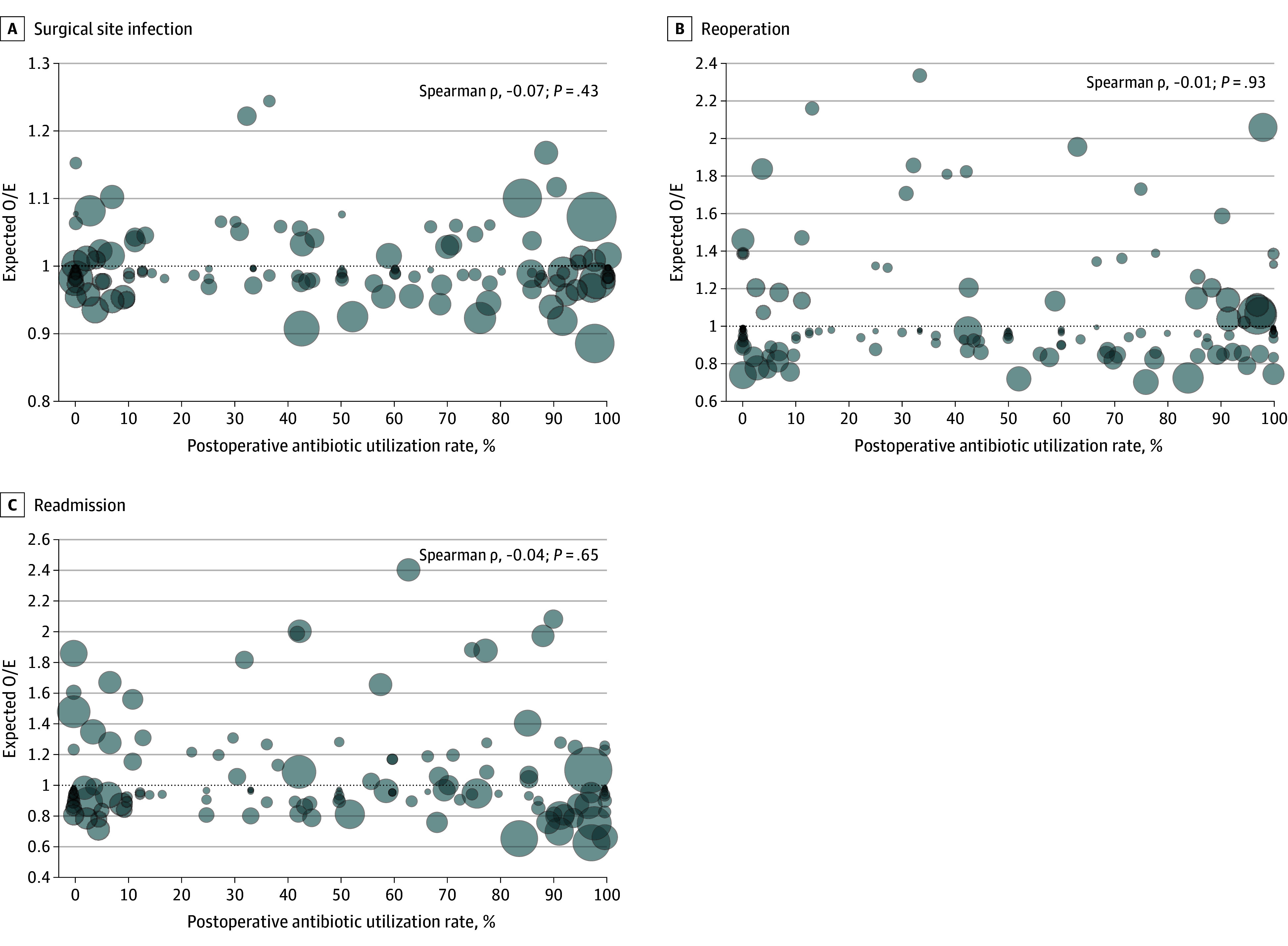
Association Between Hospital-Level Rates of Postoperative Antimicrobial Prophylaxis Use and Adjusted Observed to Expected (O/E) Surgical Site Infection Rate Ratios at 141 Hospitals Participating in the National Surgical Quality Improvement Program–Pediatric Hospital rates of each outcome were adjusted for patient characteristics (age, race or ethnicity, American Society of Anesthesiologists class, body mass index, asthma, chronic lung disease, cardiac risk factors), and surrogates for procedural complexity (operative time, *Current Procedural Technology* code, diagnosis) using mixed-effects logistic regression with a random effect for hospital. Bubble area reflects case volume at the hospital during the study period, and the dotted line is the fitted line from regression.

In the hospital-level analysis, O/E ratios for SSI rates ranged from 0.88 to 1.24 among hospitals. No correlation was found between hospital-level rates of postoperative antibiotic use and adjusted O/E SSI rate ratios (Spearman ρ, −0.07; *P* = .43) (Figure 4) or between hospital-level median duration of postoperative antibiotic use and adjusted O/E SSI rate ratios (Spearman ρ, −0.14; *P* = .09).

### Reoperation

The overall 30-day postoperative reoperation rate was 1.7% (60 of 3552 patients). Of the 60 reoperations, 9 were related to an SSI (0.25%), and the remainder of reoperations were related to noninfectious complications (eg, pneumothorax, pleural effusion, and bar displacement). Of the 9 reoperations related to an SSI, 4 (44.0%) occurred in patients who received postoperative prophylaxis. The median (IQR) time to reoperation was 14 (3-24) days.

Before matching, reoperation rates were similar for children who received postoperative antibiotics (32 of 1949 [1.6%]) compared with those who did not (28 of 1603 [1.8%]) (OR 0.97; 95% CI, 0.54- 1.72). In the propensity-matched cohort, the overall reoperation rate was 1.6%, and reoperation rates were similar in children who received postoperative antibiotics (25 of 1584 [1.6%]) compared with those who did not (27 of 1584 [1.7%]) (AOR, 0.98; 95% CI, 0.52-1.83) (Figure 3).

In the hospital-level analysis, O/E ratios for reoperation rates ranged from 0.70 to 2.33 among hospitals. No correlation was found between hospital-level rates of postoperative antibiotic use and adjusted O/E reoperation rate ratios (Spearman ρ, −0.01; *P* = .93; Figure 4) or between median hospital-level duration of postoperative antibiotic use and adjusted O/E reoperation rate ratios (Spearman ρ, −0.07; *P* = .42).

### Hospital Readmission

The overall postoperative readmission rate was 3.2% (112 of 3552). Of the 112 readmissions, 20 (0.6% of all patients) were related to an SSI. The remainder of the readmissions were related to noninfectious, procedural complications (eg, chest pain, pneumothorax, pleural effusion, and bar displacement) or were unrelated to the procedure (eg, gastritis, constipation, and emesis). Of the 20 patients requiring readmission due to an SSI, 8 (40.0%) had received postoperative prophylaxis. The median (IQR) time to readmission was 13.5 (6-23) days.

Before matching, readmission rates were similar in children who received postoperative antibiotics (55 of 1949 [2.8%]) compared with those who did not (57 of 1603 [3.6%]) (OR, 0.84; 95% CI, 0.55-1.29). In the propensity-matched cohort, the overall readmission rate was 3.1%, and admission rates were similar in children who received postoperative antibiotics (41 of 1584 [2.6%]) compared with those who did not (57 of 1584 [3.6%]) (AOR, 0.81; 95% CI, 0.48-1.34) (Figure 3).

In the hospital-level analysis, O/E ratios for readmission rates ranged from 0.62 to 2.41. No correlation was found between hospital-level rates of postoperative antibiotic use and O/E readmission rate ratios (Spearman ρ, −0.03; *P* = .65) (Figure 4) or between median hospital-level duration of postoperative antibiotic use and adjusted O/E readmission rate ratios (Spearman ρ, −0.05; *P* = .58).

## Discussion

In this multicenter cohort study of 3552 pediatric patients undergoing minimally invasive repair of pectus excavatum at 141 hospitals, postoperative continuation of antimicrobial prophylaxis was not associated with improved clinical outcomes. SSI rates were comparable between patients who did and did not receive postoperative antibiotics, and no hospital-level correlation was observed between the rate of postoperative antibiotic use and institutional SSI rates. Furthermore, use of postoperative prophylaxis did not confer a reduction in 30-day reoperation or hospital readmission rates.

To our knowledge, this study is the first multicenter analysis to evaluate the association between postoperative antibiotic use and clinical outcomes in pediatric patients undergoing minimally invasive pectus excavatum repair. Prior evidence has been limited to a single-center study involving 781 patients, which found no difference in SSI rates between children receiving less than 48 hours vs more than 48 hours of postoperative antibiotics.^12^ However, that analysis did not compare outcomes between children who did and did not receive any postoperative antibiotics, and its comparative groups arguably lacked clinical justification, as no current guidelines recommend routine continuation of antibiotics for 48 hours postoperatively.

The present study provides a more methodologically rigorous and generalizable assessment, leveraging a multicenter design and a substantially larger sample size. Unlike prior investigations that are limited by variability in outcome definitions, NSQIP-Pediatric data are collected using standardized definitions and a rigorously validated abstraction process. Consistency is maintained through regular audits, abstractor recertification, and support from the American College of Surgeons. Furthermore, the application of propensity score matching improves internal validity by minimizing confounding through balanced covariate distribution across treatment groups. Finally, the inclusion of a complementary hospital-level analysis strengthens the findings by demonstrating the lack of association between postoperative antibiotic use and SSI rates across institutions with widely varying prescribing practices.

This study has important implications for antimicrobial stewardship in the context of minimally invasive pectus excavatum repair in children. The unnecessary use of antibiotics is associated with adverse effects including *Clostridioides difficile* colitis, nephrotoxicity, and the promotion of antimicrobial resistance.^13,14^ Our findings show that SSIs (and SSI-related readmissions and reoperations) were infrequent regardless of whether postoperative prophylaxis was administered, suggesting that the potential harms of continued antibiotic use likely outweigh any marginal benefit in infection prevention for this population. Given that more than half of patients in this cohort received postoperative antibiotics, discontinuing this practice could lead to a substantial reduction in antibiotic exposure and represent a meaningful advance in antimicrobial stewardship within pediatric surgery.

### Limitations

The findings of this study should be interpreted in light of several limitations. First, the retrospective nature of the analysis introduces potential for misclassification due to errors in procedural or diagnostic coding despite the use of standardized NSQIP-Pediatric data collection methods and robust quality assurance processes. Second, unmeasured confounding may persist; for instance, patients with greater medical complexity or comorbidities related to pectus excavatum may have been more likely to receive postoperative antibiotics. To mitigate this potential bias, we used propensity score matching and conducted a complementary hospital-level analysis across a broad range of antibiotic utilization practices. Third, postoperative SSIs, reoperations, and readmissions are rare in this population, which may limit the statistical power to detect small differences in event rates. However, the observed absolute difference in SSI rates between matched groups was only 0.2%, suggesting that any undetected differences are likely to be of limited clinical relevance. Data on postoperative prophylaxis were not available for patients with coexisting oncologic or hematologic conditions, limiting the generalizability of our findings to this subgroup. Additionally, because the analysis was conducted using data from NSQIP-Pediatric–participating hospitals, the results may not be generalizable to institutions outside this hospital cohort.

## Conclusions

Although causality cannot be established due to the nonrandomized design of this study, this large multicenter cohort analysis found that use of postoperative antimicrobial prophylaxis was not associated with improved clinical outcomes in children undergoing minimally invasive pectus excavatum repair. Given that this procedure accounts for the highest relative burden of potentially avoidable postoperative antibiotic days in pediatric general surgery, reducing postoperative prophylaxis in this cohort could make a substantial impact in advancing antimicrobial stewardship.

## Notes

Supplement 1. **eFigure.** Distribution of propensity scores in the unmatched and matched cohorts by postoperative prophylaxis use

Supplement 2.
Data Sharing Statement
