# Supplementary material for: Postoperative Antimicrobial Prophylaxis Use and Outcomes in Pectus Excavatum Repair
Source: JAMA Netw Open. 2025 Sep 4;8(9):e2530449. doi: 10.1001/jamanetworkopen.2025.30449 (PMC12411969; doi:10.1001/jamanetworkopen.2025.30449)
Supplement: Supplement 1. — eFigure. Distribution of propensity scores in the unmatched and matched cohorts by postoperative prophylaxis use [file jamanetwopen-e2530449-s001.pdf]

Supplemental Online Content

McKie KA, Moturo A, Graham DA, Coleman M, Huang R, Grant C. Saito JM, Hall BL, Cina RA, Newland JG, Goretsky MJ, Ko C, Rangel SJ. Postoperative antimicrobial Prophylaxis Use and Outcomes in Pectus Excavatum Repair. *JAMA Netw Open* 2025;8(9): e2530449. doi: 10.1001/jamanetworkopen.2025.30449

eFigure. Distribution of propensity scores in the unmatched and matched cohorts by postoperative prophylaxis use

This supplemental material has been provided by the authors to give readers additional information about their work.

eFigure 1: Distribution of propensity scores in the unmatched and matched cohorts by postoperative prophylaxis use

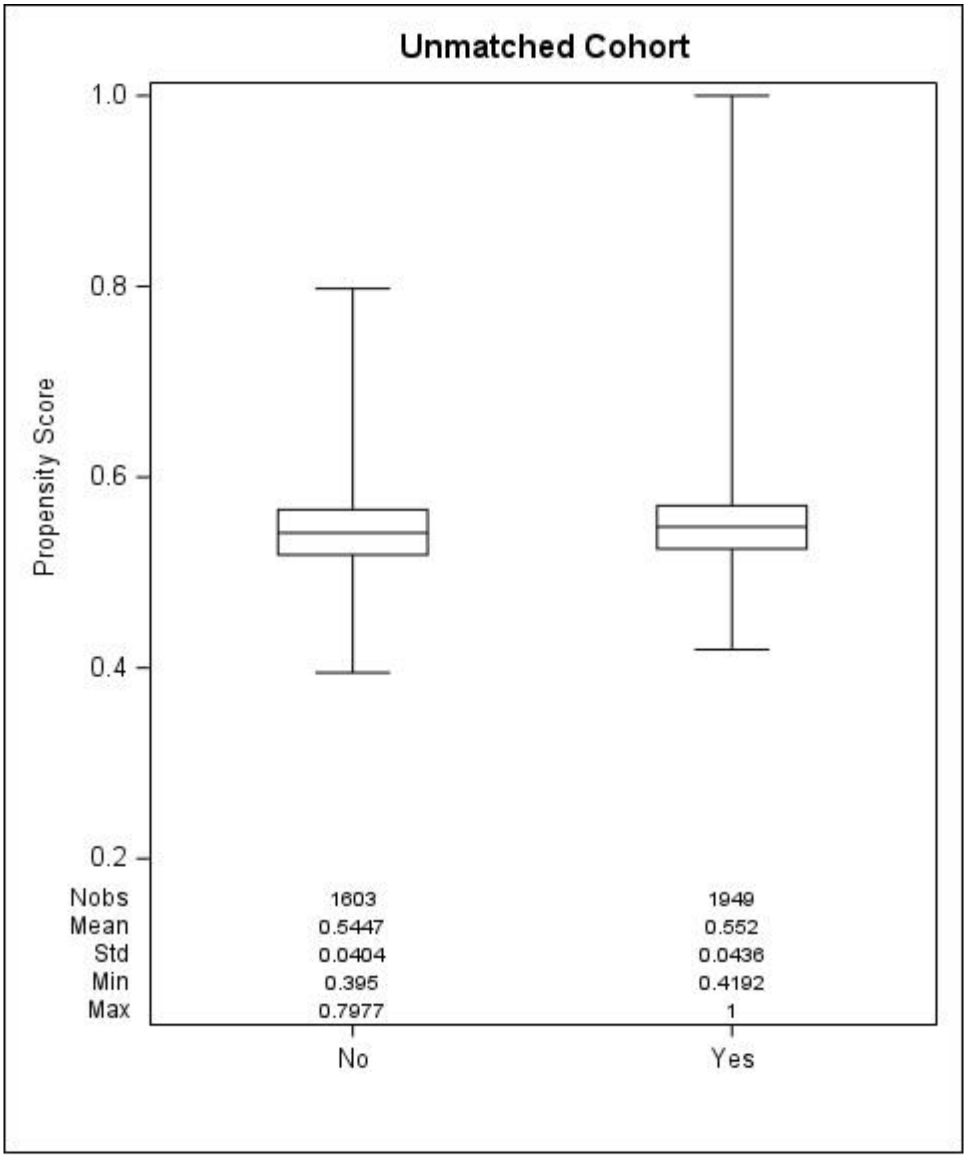

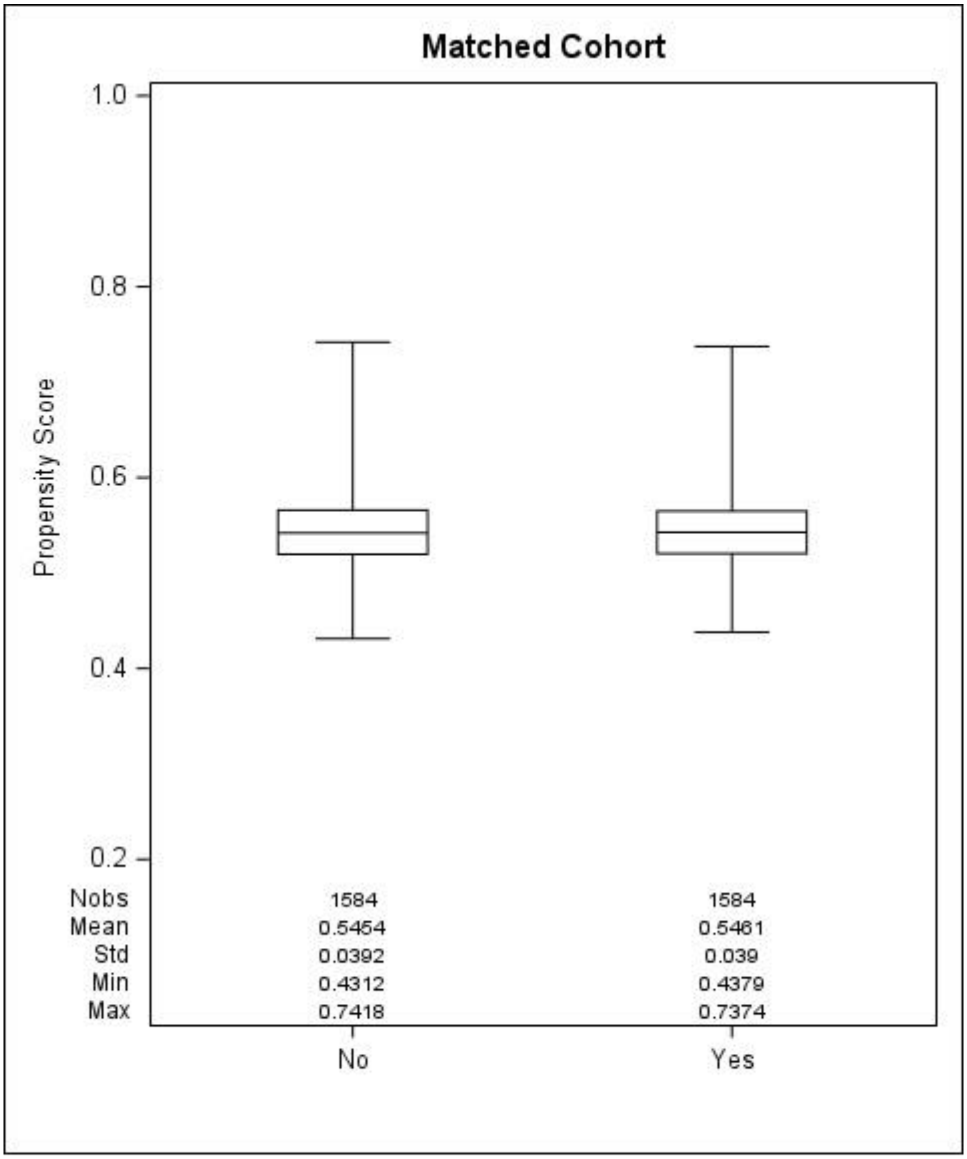
Matched Cohort
1.0
0.8
0.6
0.4
0.2
Propensity Score
Nobs
Mean
Std
Min
Max
1584
0.5454
0.0392
0.4312
0.7418
1584
0.5461
0.039
0.4379
0.7374
No
Yes
