# Supplementary material for: Postoperative Antimicrobial Prophylaxis Use and Outcomes in Pectus Excavatum Repair
Source: JAMA Netw Open. 2025 Sep 4;8(9):e2530449. doi: 10.1001/jamanetworkopen.2025.30449 (PMC12411969; doi:10.1001/jamanetworkopen.2025.30449)
Supplement: Supplement 2. — Data Sharing Statement [file jamanetwopen-e2530449-s002.pdf]

Data Sharing Statement

Data
Data available: No

Additional Information
Explanation for why data not available: American College of Surgeons NSQIP data is available at participating institutions but is not publicly available.
